# Application of Machine Learning in Material Synthesis and Property Prediction

**DOI:** 10.3390/ma16175977

**Published:** 2023-08-31

**Authors:** Guannan Huang, Yani Guo, Ye Chen, Zhengwei Nie

**Affiliations:** School of Mechanical and Power Engineering, Nanjing Tech University, Nanjing 211816, China; i35653184@163.com (G.H.); guo17793470078@163.com (Y.G.); chenye@njtech.edu.cn (Y.C.)

**Keywords:** machine learning, material screening, property prediction, material synthesis, artificial intelligence

## Abstract

Material innovation plays a very important role in technological progress and industrial development. Traditional experimental exploration and numerical simulation often require considerable time and resources. A new approach is urgently needed to accelerate the discovery and exploration of new materials. Machine learning can greatly reduce computational costs, shorten the development cycle, and improve computational accuracy. It has become one of the most promising research approaches in the process of novel material screening and material property prediction. In recent years, machine learning has been widely used in many fields of research, such as superconductivity, thermoelectrics, photovoltaics, catalysis, and high-entropy alloys. In this review, the basic principles of machine learning are briefly outlined. Several commonly used algorithms in machine learning models and their primary applications are then introduced. The research progress of machine learning in predicting material properties and guiding material synthesis is discussed. Finally, a future outlook on machine learning in the materials science field is presented.

## 1. Introduction

New materials have become the cornerstone of scientific and technological development. Discovering materials with targeted properties, especially nanomaterials, has always been a hotspot in science [1,2]. At present, the research and development of new materials mainly relies on researchers’ intuitive judgment of materials and empirical trial-and-error methods, which are not only inefficient but also often require a certain level of experience and luck to obtain the target materials. At the same time, methods based on density functional theory (DFT) are widely used in the research and development of novel materials. Since their initial development, DFT methods have evolved from limited calculations that provide approximate results to increasingly accurate and predictable methods. These methods have made important contributions in a variety of fields, such as materials discovery and design, drug design, solar cells, and hydrolytic materials [3]. The accuracy of these methods, however, is limited in fast calculations. To obtain high-accuracy results, the computational volume often has to be much higher, which is difficult to exploit efficiently in the research and development of new materials. In this context, artificial intelligence (AI) is becoming highly popular with researchers as a means of accelerating the development of innovative materials. A subfield of AI that has grown rapidly in recent years is machine learning (ML). ML applications are built on statistical algorithms. ML performs similarly to researchers’ performance [4]. Because of its powerful data processing capability and relatively low research threshold, ML can effectively reduce human and material costs in the process of novel material development and shorten the research and development cycle. By replacing or collaborating with traditional experiments and computational simulations, ML could be employed to analyze material structures and predict material properties, enabling the development of novel functional materials more efficiently and accurately. As a result, ML has become one of the most crucial methods for replacing traditional research and development. In the recent past, researchers in different fields, including computer scientists and experts in AI algorithms, have used this approach extensively, greatly contributing to the development of ML techniques [5]. ML is now widely utilized in fields such as natural language understanding, non-monotonic reasoning, machine vision, and pattern recognition [6].

**Figure 1:**
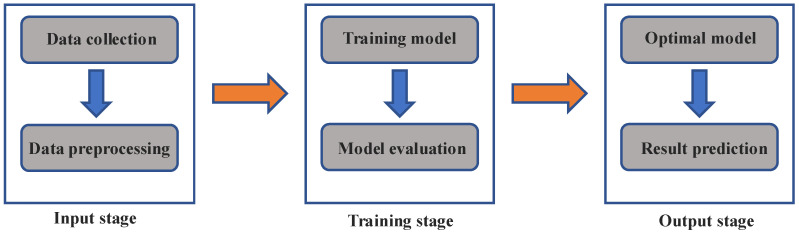
An example of an ML workflow.

The basic principle of ML is to learn (or guess) general patterns from a limited amount of training data and use these patterns to make predictions on unknown data. Figure 1 shows an ML workflow example. ML has been used to detect the solubility of C_60_ in materials science as early as the last century [7]. It is now used to discover novel materials, predict material and molecular properties, study quantum chemistry, and design drugs. The purpose of this review is to offer an overview of the employment of ML in predicting material properties and performance, guiding material synthesis, and projecting models and conclusions. This review not only provides guidance for researchers to synthesize stable and efficient materials, but also inspires their interest in the use of ML in materials research.

## 2. Data Pre-Processing

If ML models are the engines that handle various tasks, data are the fuel that drives the models. A sufficient amount of data is a prerequisite to making the model work. High-quality data enable the model to run effectively. Due to this, large amounts of data are critical to ML [8]. In general, the final ML results are directly affected by the amount and reliability of the data. This is where data pre-processing and feature engineering are beneficial. Data pre-processing and feature engineering could promote the reconstruction of datasets so that computers could more easily understand the physicochemical relationships of materials, detect material properties, and build prediction models [9].

### 2.1. Data Collection and Cleaning

#### 2.1.1. Data Collection

In ML, the size and quality of the training dataset employed for learning could significantly affect the accuracy of a predictive model. Therefore, training datasets need to be collected or created carefully. In general, training data can be gathered in three ways. Obtaining data from the published literature is the first method. The data obtained in this way could be more relevant and provide a direction for synthesis and application [10]. Second, high-throughput computations or experiments can be used to obtain data. It should be noted that, in some cases, these data may be incomplete, inconsistent, or even spurious [11]. The third method is to obtain data from open databases available on repository websites. The Materials Genome Initiative, initiated by the United States in 2011, emphasizes the importance of massive data in the development of materials science, which encourages the development of high-quality material databases [12]. With the continuous development of theoretical and experimental research, data generated from experiments and computational simulations, including failure data, have been integrated into databases [13]. These databases are based on the concept of material data sharing, which greatly simplifies the process of obtaining material information. Table 1 introduces some commonly used methods for collecting data from publicly available databases. For instance, Zhou et al. [14] developed an ML-based approach to predict cathode materials for Zn-ion batteries with high capacity and high voltage. They screened over 130,000 inorganic materials from the materials project database and applied a crystal graph convolutional-neural-network-based ML approach with data from the Automatic Flow (AFLOW) database. This resulted in the prediction of approximately 80 cathode materials, with 10 of them being experimentally discovered previously and agreeing well with the observed measurements. Additionally, approximately 70 new promising candidates were predicted for further experimental validation.

#### 2.1.2. Data Cleaning

When collecting raw data, unprocessed datasets are difficult to analyze and sometimes become useless, as they tend to be inconsistent, missing, and noisy. Before using those datasets, quality must be maintained. Data cleaning is an operation performed on the existing data to remove anomalies and obtain the data collection, which is an accurate and unique representation of the mini world. It involves eliminating errors, resolving inconsistencies, and transforming the data into a uniform format [15]. Data cleaning is an enormous task achieved by smoothing noise, completing missing values, correcting inconsistencies, and identifying outliers in data. The common methods for filling in missing values are as follows: fill in missing values manually; fill in missing values with a global constant; fill in missing values with the average value of attributes; fill in corresponding missing values with the average value of attributes of the same type as the given tuple; and fill in missing values with the most likely value. The commonly used methods for smoothing noise are binning, regression, and clustering [10]. Binning is employed to handle noisy data. In this approach, the data are sorted, and then values are partitioned by equal-frequency bins where values are put into an equal number of bins. Regression involves predicting unknown data from known data and fitting it using a function. The two types of regression techniques are linear and multiple linear. Linear regression uses a known value to predict an unknown value, fitting the relationship between the two values with a straight line. To reduce outliers, clustering can be implemented. Clustering refers to grouping data points with similar properties into clusters. By categorizing outliers as points outside these clusters, they could be easily identified and minimized in the dataset [16,17,18]. Data cleaning can effectively improve the model’s prediction accuracy. Liu et al. [19] discussed the prediction of protein–protein interaction sites using ML-based computational approaches. The authors proposed a method that improves prediction performance by addressing the class imbalance issue in protein–protein interaction site prediction. They operated a data-cleaning procedure to remove marginal targets from majority samples and a post-filtering procedure to reduce false-positive predictions. The proposed method was tested on benchmark datasets and showed competitive performance compared to existing predictors.

### 2.2. Feature Engineering

A key part of the data preparation phase in ML is feature engineering. It extracts features (also known as descriptors) from the raw data and transforms the features into a format suitable for ML models. The selection of features is critical for building ML models and could even determine the upper limit of overall model performance [20]. In feature selection, different parameters could be operated as features for chemical and material structures (and their properties), e.g., electronic properties (band gap, dielectric constant, work function, electron density, and electron affinity) and crystal features (translation vectors, fractional coordinates of atoms, radial distribution functions, and Voronoi tessellations of atomic positions). It is worth noting that rational feature selection is often expensive and difficult [11]. In past studies, feature selection has typically had to be performed manually. However, the limitations of manual feature engineering prevented the selection of the most representative features in most cases. Over the last few years, the employment of automated feature engineering has become increasingly widespread. It automatically constructs brand new candidate features from data and selects the most suitable features for model training, which could solve the dilemma faced by manual feature engineering.

**Figure 2:**
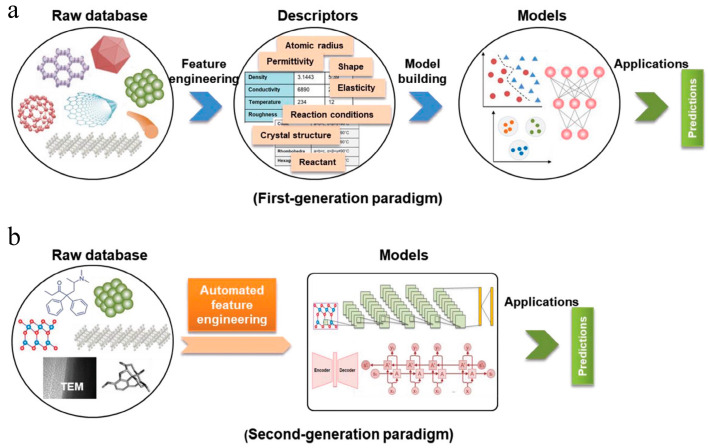
Evolution of the ML workflow in nanomaterial discovery and design. (**a**) First-generation approach. In this paradigm, there are two main steps: feature engineering from raw database to descriptors and model building from descriptors to target model. (**b**) Second-generation approach. The key characteristic that distinguishes this approach from the first-generation approach is eliminating human-expert feature engineering, which can directly learn from raw nanomaterials. Reproduced with permission from [21].

Wang et al. [21] utilized automated feature engineering for the development of nanomaterials. Automated feature engineering uses deep learning algorithms to automatically develop a set of features that are relevant to the desired output. As a result, non-experts could select features much more easily, which would greatly reduce the use of expertise in training models. The variation in feature engineering in the design of nanomaterials can be observed in Figure 2.

## 3. Classification of ML and Algorithms

Once sufficient training data are selected, models can be built for the development of novel materials. Choosing an appropriate algorithm for a training model is essential for making accurate predictions. Based on the type of processed data, ML can be classified as supervised learning, unsupervised learning, semi-supervised learning, and reinforcement learning. For supervised learning, the input training data are labeled. After optimizing the model with ML, a predictable output value for a new input value could be acquired. In contrast, the input training data are unlabeled in unsupervised learning. Using an algorithm, the unlabeled training set is trained to find potential features. As for semi-supervised learning, the input training data are partially labeled. Reinforcement learning occurs when the training object interacts with the environment, obtaining feedback from the environment and adjusting its strategy to accomplish a specific goal or to maximize the benefit of a behavior [22]. Next, a brief description of several commonly utilized ML algorithms is given.

### 3.1. Shallow Learning

Shallow learning usually has no hidden layer or only one hidden layer [23]. The approaches include decision tree (DT), K-nearest neighbor (KNN), support vector machine (SVM) [24], random forest (RF), and artificial neural network (ANN). Shallow learning has produced satisfactory results in various areas of materials science. In this section, some algorithms for shallow learning are presented, some applications in materials science are summarized, and the ML model used by the researchers is demonstrated.

#### 3.1.1. KNN

**Figure 3:**
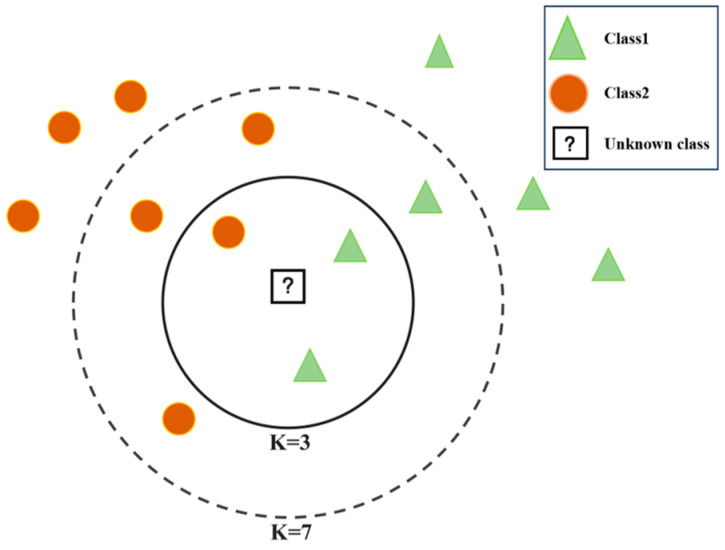
Schematic of a typical KNN algorithm.

The KNN algorithm was first proposed by Cover and Hart [25]. The KNN classification is one of the most basic and simplest classification methods. It should be considered for classification studies when little or no data distribution experience is available [26]. The principle of the KNN algorithm is that if most of the most similar K samples in the feature space (i.e., the nearest samples in the feature space) belong to a certain category, the sample also belongs to this category. Figure 3 shows a schematic of a typical KNN algorithm. For an unknown target, when K takes 3, the target is classified into class 1; when K takes 7, the target is classified into class 2. According to this method, the sample’s category is determined by its proximity to one or more nearby samples. The KNN algorithm itself is simple and effective, easy to understand, and straightforward to implement. Since it does not require prediction parameters or training, the KNN algorithm is suitable for time classifications, especially for multimodals (i.e., objects with multiple categories). Recently, KNN algorithms have been widely utilized in text classification, pattern recognition, image processing, and materials science. Sharma et al. [27] employed the KNN algorithm to predict the dynamic fracture toughness of glass-filled polymer composites. The dynamic modulus of elasticity, aspect ratio, and volume fraction of glass particles were used as independent model parameters. The proposed KNN model predicted the fracture behavior of the composites with an accuracy of 96%. It is also possible to extend their model to predict other material properties.

The drawback of the KNN algorithm is that as the amount of data increase, the computational complexity of the KNN increases accordingly. This is because the KNN algorithm needs to calculate both training data and test data for each classification or regression. If there are a large amount of data, the computing power required would be greatly increased. In addition, the randomness of training data also affects the performance of the KNN algorithm [28].

#### 3.1.2. DT

**Figure 4:**
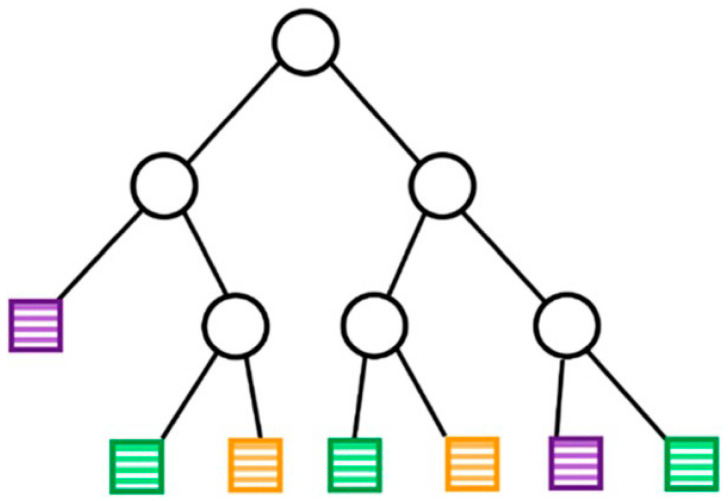
Diagram of a DT. The circles and squares indicate internal nodes and leaf nodes, respectively. Different colors represent different classes. Reproduced with permission from [11].

A DT is a typical classification method. The earliest DT algorithm was the concept learning system proposed by Hunt [29]. The most influential DT algorithms are ID3 [30] and C4.5 [31], which were proposed by Quinlan in 1986 and 1993, respectively. DTs classify training data by different features, aiming to correctly categorize instances. A DT model consists of internal decision nodes and leaf nodes. Each internal node splits the instance space into two or more subspaces according to a certain discrete function of the input attribute values, and each leaf node is assigned to one class representing the most appropriate target value [32]. Chen et al. [11] presented the structure of a typical DT, as shown in Figure 4. A typical decision tree algorithm consists of three main steps: feature selection, decision tree generation, and pruning. The purpose of pruning is to minimize the structural risk of the model by optimizing the loss function and weighing the model’s complexity and accuracy. Liu et al. [33] developed a DT model for predicting the residual tensile strength and modulus of pultruded-fiber-reinforced polymer (FRP) composites. Using an existing database, 746 data points were collected for training. The accuracy of the model was verified experimentally. The significance of all attributes of the input data was also quantitatively analyzed by the model. The proposed DT model provides a new method for predicting the long-term degradation of FRP composites subjected to environmental influences.

The RF algorithm consists of multiple DTs. In RFs, each tree casts a unit vote for the most popular class, and then combining these votes obtains the final sort result. RFs possess high classification accuracy [34]. It would, however, take a great deal of space and time to train an RF with many DTs. Compared with DTs, the calculation costs of RFs would also increase significantly. In this regard, RFs and DTs should be selected based on the actual situation.

#### 3.1.3. ANN

The concept of an ANN was introduced by McCulloch and Pitts [35]. An ANN is a complex network structure that is formed by a large number of nodes (neurons) connected to each other. It is a kind of abstraction, simplification, and simulation of the organization and operation mechanism of the human brain. Each node in an ANN represents a specific output function, i.e., the activation function. Each connection between any two nodes represents a weighted value for the signal passing through that connection, which is equivalent to the memory of the ANN. The network’s connection mode, the value of the weights, and the excitation function all have an effect on its output [36]. As a major soft-computing technology, ANNs have been extensively studied and applied in recent decades [37].

**Figure 5:**
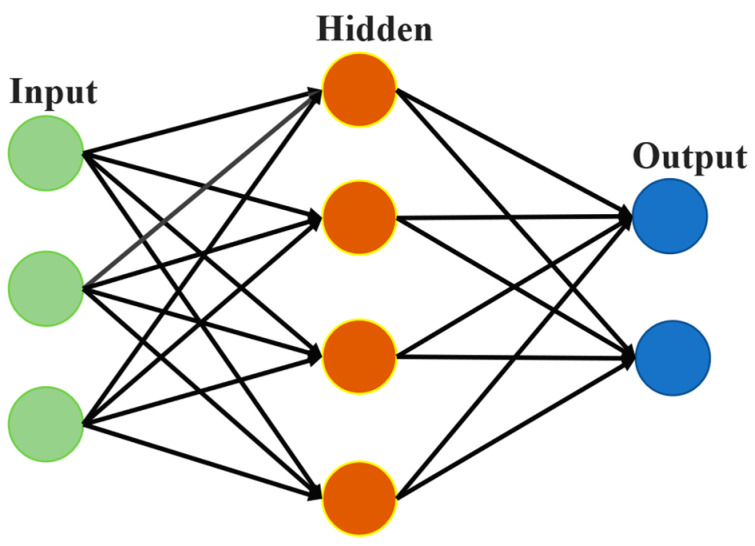
Diagram of a typical ANN.

The structure of a typical ANN is shown in Figure 5. Its nodes are generally divided into three categories: input, hidden, and output. The input nodes represent the information received from the input data. The output nodes are utilized to store the results of the data processing. The nodes between the input and output nodes are so-called hidden nodes. Different types of nodes in an ANN are distributed in multiple layers. The nodes on different layers could be connected by lines, which correspond to synapses in neural structures, representing a nonlinear mapping. The learning process of an ANN is to continuously optimize the whole network model by correcting the weights of nodes in each layer with training data [38].

A variety of ANN models and their variants have been developed. The variants include back-propagation networks, perceptrons, self-organizing mappings, Hopfield networks, and Boltzmann machines. ANNs have been applied to drive the synthesis of a wide range of functional materials, such as shape memory alloys [39], hyperelastic materials [40], and high-entropy alloys (HEAs) [41]. Table 2 illustrates the application of the afore-mentioned algorithms.

**Table 2:** Some applications of shallow learning in materials science.

Researchers	Algorithms	Purposes
Sharma et al. [42]	KNN	Predict the fracture toughness of silica-filled epoxy composites.
Kumar et al. [43]	KNN	Predict surface roughness in the micro-plasma transfer arc metal additive manufacturing (μ-PTAMAM) process.
Jalali et al. [44]	KNN (Figure 6a)	Predict phases in HEAs.
Wang et al. [45]	SVM	Achieve rapid detection of transformer winding materials.
Martinez et al. [46]	SVM and ANN	Predict the fracture life of martensitic steels under high-temperature creep conditions.
Ahmad et al. [47]	Adaptive boosting, RF, and DT (Figure 6b)	Predict the compressive strength of concrete at high temperatures.
Sun et al. [48]	Gradient boosted regression tree (GBRT) and RF	Evaluate the strength of coal–grout materials.
Samadia et al. [49]	GBRT	Predict the higher heating value (HHV) of biomass materials based on proximate analysis.
Shahmansouri et al. [50]	ANN (Figure 6c)	Predict the compressive strength of eco-friendly geopolymer concrete incorporating silica fume and natural zeolite.
Liu et al. [51]	ANN	Development of a predictive model for the chloride diffusion coefficient in concrete.

**Figure 6:**
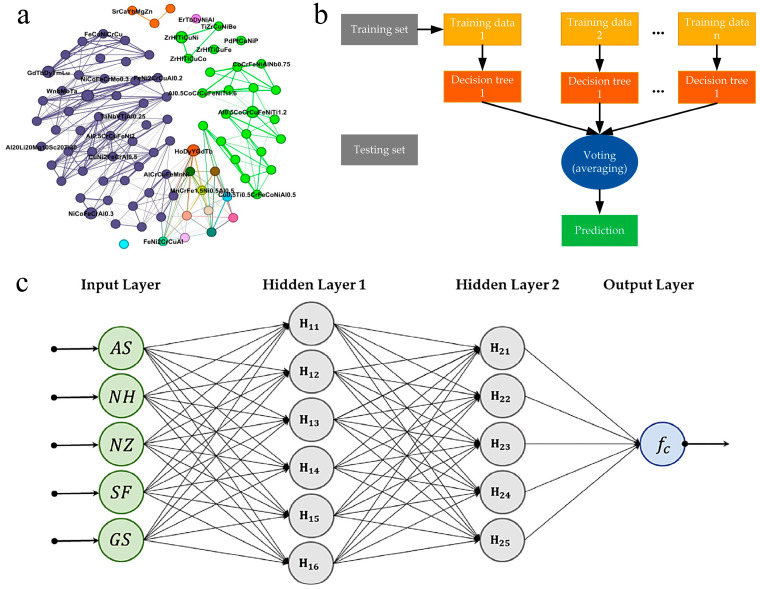
(**a**) A portion of the HEA interaction network with Fruchterman Reingold layout, adapted with permission from [44]. (**b**) Schematic illustration of an RF structure, adapted with permission from [47]. (**c**) A multi-layer neural network model layout, adapted with permission from [50].

### 3.2. Deep Learning

Hinton et al. [52] first proposed the concept of deep learning. The unsupervised greedy training layer-by-layer algorithm based on deep degree nets was designed to solve optimization problems related to deep structures. Similar to an ANN, deep learning is a multilayer neural network [53].

#### 3.2.1. Overview of Deep Learning

Deep learning can be considered a subset of ML. The idea of deep learning is derived from multilayer ANNs. The learning process of deep learning exhibits depth to some extent because of the multilayer structure of ANNs. In each hidden layer, neurons receive input signals from other neurons, combine them with their internal state, and produce output signals. The connections between neurons have weights assigned to them, forming the overall layer of a neural network. The learning process involves adapting the network by adjusting the weights of the connections to minimize output errors. Deep learning, with its self-adapting architecture, reduces the need for feature engineering and could identify and work around defects that may be difficult to detect in other techniques [5]. Instead, the algorithm adjusts itself in continuous learning and independently selects suitable features. This could be viewed as a major advancement in ML. While traditional ML models may be more accurate with small data, deep learning models tend to be more reliable when big data is available. Deep neural networks (DNNs) with multiple hidden layers have higher learning capacity, allowing them to saturate accuracy gains compared to traditional models. Although training neural networks is computationally expensive, once trained, deep learning can make very fast predictions. This one-time training cost is outweighed by the speed of subsequent predictions [54]. After years of development, a variety of deep learning models have been produced, mainly including stacked autoencoders [55], deep belief networks (DBNs) [56], deep Boltzmann machines (DBMs) [57], DNNs [58], and convolutional neural networks (CNNs) [59]. Deep learning techniques are widely utilized in speech recognition, visual object recognition, object detection, drug discovery, and genomics [60]. They are also some of the fastest-growing and most adaptable techniques ever developed in materials science.

Additionally, deep learning faces the dilemma of how to effectively process large amounts of complex data. In practical applications, building suitable deep learning models is increasingly challenging. Although deep learning is not yet fully mature and has many problems to solve, it has shown a strong learning capability. Throughout the future, deep learning is expected to remain a key research focus in AI.

#### 3.2.2. Applications of Deep Learning

**Figure 7:**
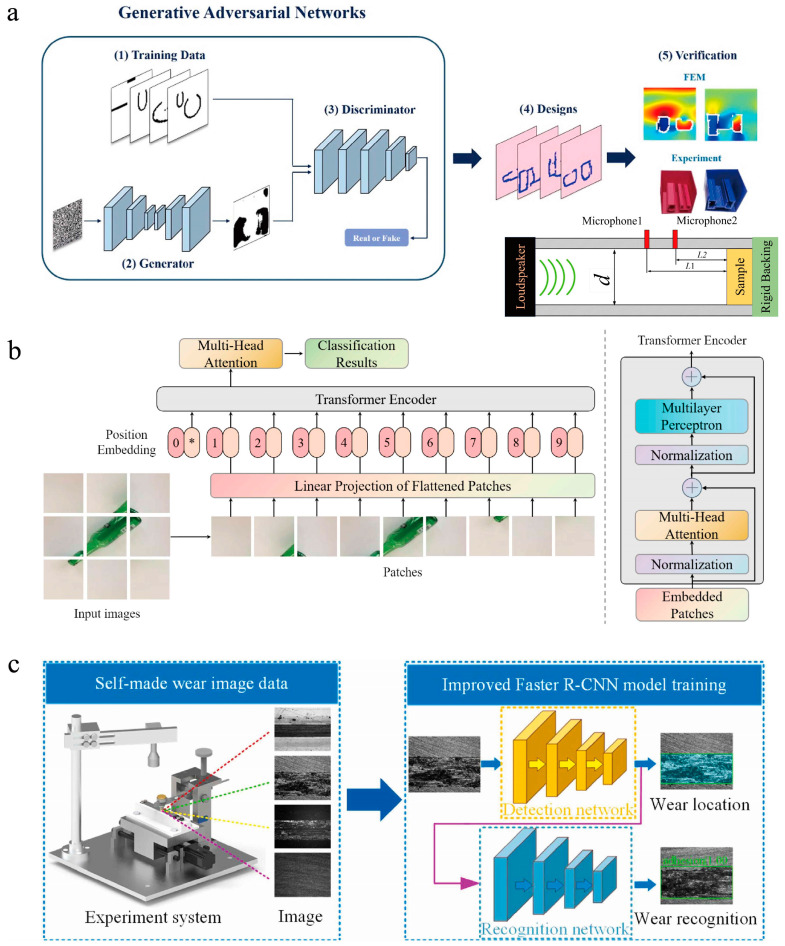
Some deep learning algorithm structures. (**a**) Schematic illustration of the design procedures of metaporous materials with GANs, adapted with permission from [65]. (**b**) Structure of a vision transformer, adapted with permission from [67]. (**c**) Illustration of the concept of using image identification based on the improved Faster R-CNN model to identify wear, adapted with permission from [69].

Deep learning has been widely applied in materials science due to its excellent performance. Based on industrial data, Wu et al. [61] investigated the impact energy prediction model of low-carbon steel. A three-layer neural network, extreme learning machine, and DNN were compared with different activation functions, structure parameters, and training functions. Bayesian optimization was employed to determine the optimal hyper-parameters of the DNN. The model with the highest performance was applied to investigate the importance of process parameter variables on the impact energy of low-carbon steel. The results showed that the DNN obtained better prediction results than those of a shallow neural network because the multiple hidden layers improved the learning ability of the model. Sun et al. [62] applied deep learning to rapidly predict the photovoltaic properties of organic photovoltaic materials, with a prediction accuracy up to 91%. Konno et al. [63] reported a deep learning algorithm for discovering novel superconductors. The prediction accuracy of their ML model for material superconductivity was as high as 62%. Employing the ML model, the authors found two superconductors that were not in the database and found Fe-based high-temperature superconductors (discovered in 2008) in the training data before 2008. These results pave the way for the discovery of new high-temperature superconductors. Li et al. [64] explored a correlated deep learning framework consisting of three recurrent neural networks (RNNs) to efficiently generate new energetic molecules with high detonation velocity in the low data regime. They utilized data augmentation by fragment shuffling of 303 energetic compounds to pretrain the RNN and then fine-tuned it using the 303 compounds to produce molecules similar to the energetic compounds. They also employed a simplified molecular input line entry (SMILE) system coupled with pretrained knowledge to build an RNN-based prediction model for screening molecules with high detonation velocity. Their strategy performed comparably to transfer learning based on an existing big database. Quantum mechanics calculations confirmed that 35 new molecules have higher detonation velocity and lower synthetic accessibility than the classic explosive hexogen, with three novel molecules comparable to caged China Lake Compound No. 20 in detonation velocity. Zhang et al. [65] utilized generative adversarial networks (GANs) to design metaporous materials for sound absorption (Figure 7a). The researchers trained the GANs using numerically prepared data and successfully developed designs with high-standard broadband absorption performance. The GANs accelerated the design process by hundreds of times, allowing for instantaneous multiple solutions. The GANs also demonstrated the ability to generate creative configurations and rich local features. This work highlighted the potential of ML in guiding the design and optimization process for materials and opened up new possibilities for interdisciplinary research in AI and materials. Unni et al. [66] introduced a deep convolutional mixture density network (MDN) approach for the inverse design of layered photonic structures. The MDN modeled the design parameters as multimodal probability distributions, allowing for convergence in cases of nonuniqueness without sacrificing degenerate solutions. The MDN was applied to the inverse design of two types of multilayer photonic structures consisting of thin films of oxides, which present a challenge for conventional machine learning algorithms due to their large degree of nonuniqueness in their optical properties. The MDN can handle the transmission spectra of high complexity and varying illumination conditions. The shape of the probability distributions provides valuable information for postprocessing and prediction uncertainty. The MDN approach offers an effective solution to the inverse design of photonic structures with high degeneracy and spectral complexity.

The use of vision transformers, residual networks (ResNets), and region-based-CNNs (R-CNNs) on materials datasets has shown exceptional performance. Huang et al. [67] proposed a waste materials classification method based on a vision transformer model (Figure 7b). The model overcame CNN limitations by using self-attention mechanisms to allocate weights to different parts of waste images. The vision transformer achieved an accuracy rate of 96.98% by pretraining on ImageNet and fine-tuning on the TrashNet dataset. The trained model can be deployed on a cloud server and accessed through a portable device for real-time waste classification, which is convenient and efficient for resource conservation and recycling. Jiang et al. [68] explored the use of global optimization networks (GLOnets) with the ResNet architecture for the multiobjective and categorical global optimization of photonic devices. The authors demonstrated that these networks, called Res-GLOnets, could be configured to design thin-film stacks consisting of multiple material types. The Res-GLOnets can find the global optimum with faster speeds compared to conventional algorithms. The authors also showed the utility of their method for complex design tasks, such as designing incandescent light filters. Wang et al. [69] proposed an image detection method based on an improved Faster R-CNN model for wear location and wear mechanism identification (Figure 7c). They trained and tested the model using a wear image dataset produced by a self-made tribometer equipped with an imaging system. The results showed that the proposed method had a detection accuracy of more than 99%. It outperformed edge detection technology and Yolov3 target detection models in wear location and wear mechanism identification. This research contributes to the development of an innovative approach for the online and intelligent wear status detection of machinery components.

### 3.3. Materials Informatics Based on ML

**Figure 8:**
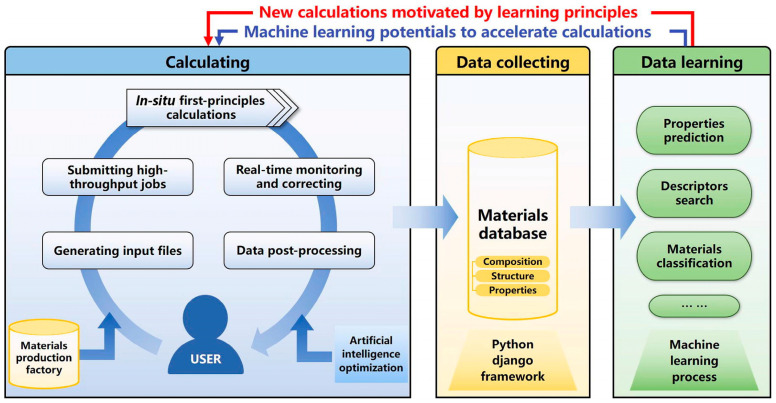
Overview of the JAMIP code framework. The program comprises three major parts based on the material data’s lifecycle: data generation (blue), data collection (yellow), and data learning (green). Reproduced with permission from [74].

Materials informatics is a study field that focuses on investigating and applying informatics techniques to materials science and engineering. Propelled partly by the Materials Genome Initiative and partly by algorithmic developments and successes of data-driven efforts in other domains, informatics strategies are beginning to take shape within materials science. Informatics strategies give rise to surrogate ML methods that can realize accurate prediction using just historical data instead of experiments or simulations/calculations. This methodology is usually composed of three distinct steps: acquisition of reliable historical data, statistical quantification of information-rich material structures, and mapping between “input” and “output”. The commonly used ML algorithms in materials informatics include regression, DT, ANN, and deep learning [70,71,72,73]. To meet the requirements of the studies of computational materials informatics, Zhao et al. [74] derived an artificial-intelligence-aided data-driven infrastructure called Jilin Artificial-intelligence aided Materials-design Integrated Package (JAMIP). The organization of JAMIP abides by the data lifecycle in computational materials informatics, from data generation to collection and learning, as shown in Figure 8. It provides tools for materials production, high-throughput calculations, data extraction and management, and ML-based data mining. The authors demonstrated the usefulness of JAMIP in exploring materials informatics in optoelectronic semiconductors, specifically halide perovskites. Hu et al. [75] proposed and developed MaterialsAtlas.org (accessed on 19 August 2023), a web-based materials informatics toolbox. The MaterialsAtlas platform includes tools for chemical validity check, formation energy and e-above-hull energy check, property prediction, screening of hypothetical materials, and utility tools. The toolbox lowers the barrier for materials scientists in data-driven exploratory materials discovery.

## 4. ML in Materials Science

### 4.1. Prediction of Material Properties

ML has gained prominence in recent years in predicting material properties due to its advantages of high generalization ability and fast computational speed. It has been successfully applied to predict the structure, adsorption, electrical, catalytic, energy storage, and thermodynamic properties of materials. The prediction results could even reach the same accuracy as high-fidelity models with low computational costs.

#### 4.1.1. Molecular Properties

In the past, it was very time consuming to predict molecular properties based on high-throughput density generalization calculations. ML allows fast and accurate prediction of the structure or properties of molecules, compounds, and materials. In materials science, solubility factors, such as Hansen and Hildebrand solubility, are critical parameters for characterizing the physical properties of various substances. Kurotani et al. [76] successfully developed a solubility prediction model with a unique ML method, the so-called in-phase DNN (ip-DNN). This algorithm started with the analysis of input data (including NMR information, refractive index, and density). The solubility was then speculated in a multi-step approach by predicting intermediate elements, such as molecular components and molecular descriptors. An intermediate regression model was also utilized to improve the accuracy of the prediction. A website dedicated to the established solubility prediction methods has also been developed, which is available free of charge. Liang et al. [77] proposed a generalized ML method based on ANNs to predict polymer compatibility (the total miscibility of polymers with each other at the molecular scale). The authors built a database by collecting data from scattered literature through natural language processing techniques. By using the proposed method, predictions could be made based on the basic molecular structure of the blended polymers and the blended compositions (as an auxiliary). This generalized approach yielded some results in illustrating polymer compatibility. A prediction accuracy of no less than 75% was achieved on a dataset containing 1400 entries in their model. Zeng et al. [78] developed an atomic table CNN that could predict the band gap and ground energy. The model accuracy exceeded that of standard DFT calculations. Furthermore, this model could accurately predict superconducting transition temperatures and distinguish between superconductors and non-superconductors. With the help of this model, 20 potential superconductor compounds with high superconducting transition temperatures were screened out.

#### 4.1.2. Band Gap

The band gap size not only determines the energy band structure of a material but also affects its electronic structure and optical properties. Recently, researchers have applied ML to forecast the band gap of various materials. Venkatraman [79] developed an algorithm for band gap prediction based on a rule-based ML framework. With descriptors derived from elemental compositions, this model accurately and quickly predicted the band gap of various materials. After testing on two independent sets, this model obtained squared correlations > 0.85, with errors smaller than those of most density generalization calculations, improving the material screening performance. Xu et al. [80] developed an ML model called support vector regression (SVR) for predicting the band gaps of polymers. They used training data obtained from DFT computations and generated descriptors using Dragon software. After feature selection, the SVR model using 16 key features achieved high accuracy in predicting polymer band gaps. The SVR model with a Gaussian kernel function performed the best, with a determination coefficient (*R*^2^) of 0.824 and a root mean square error (RMSE) of 0.485 in leave-one-out cross-validation. The authors also provided correlation analysis and sensitivity analysis to understand the relationship between the selected features and the band gaps of polymers. Several polymer samples with targeted band gaps were designed based on the analysis and validated through DFT calculations and model predictions. Espinosa et al. [81] proposed a vision-based system to predict the electronic band gaps of organic molecules using deep learning techniques. The system employed a multichannel 2D CNN and a 3D CNN to recognize and classify 2D projected images of molecular structures. The training and testing datasets used in the research were derived from the Organic Materials Database (OMDB-GAP1). The results showed that the proposed CNN model achieved a mean absolute error of 0.6780 eV and an RMSE of 0.7673 eV, outperforming other ML methods based on conventional DFT. These findings demonstrate the potential of CNN models in materials science applications using orthogonal image projections of molecules. Wang et al. [82] explored the use of ML techniques to accurately predict the band gaps of semiconductor materials. The authors applied a stacking approach, which combined the outputs of multiple baseline models, to enhance the performance of band gap regression. The effectiveness of different models was tested using a benchmark dataset and a newly established complex database. The results showed that the stacking model had the highest *R*^2^ value in both datasets, indicating its superior performance. The improvement percentages of various evaluation metrics for the stacking model compared to other baseline models range from 3.06% to 33.33%. Overall, the research demonstrated the excellent performance of the stacking approach in band gap regression. On the basis of generalized gradient approximation (GGA) band gap information of crystal structures and materials, Na et al. [83] established an ML method that used the tupleswise graph neural network (TGNN) algorithm for the accurate band gap prediction of crystalline compounds. The TGNN algorithm showed strong superiority in predicting the band gap of four different open databases. It has better accuracy for 48,835 samples of G_0_W_0_ (a widely used technique in which the self-energy is expressed as the convolution of a noninteracting Green’s function (G_0_) and a screened Coulomb interaction (W_0_) in the frequency domain) band gaps than the standard density generalized theory without high computational costs. Moreover, this model could be extended to project other valuable properties.

#### 4.1.3. Energy Storage Performance

**Figure 9:**
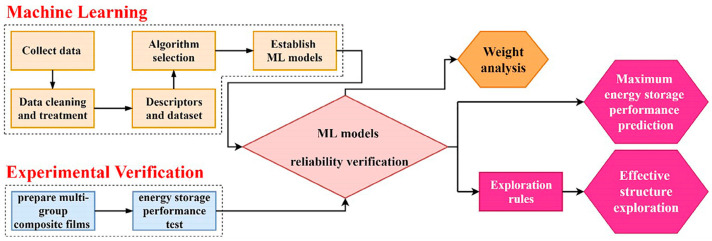
Logic diagram of predicting the maximum energy density and exploring the potential effective structure of composites through the ML method, reproduced with permission from [85].

Energy storage is a key step in determining the efficiency, stability, and reliability of power supply systems [84]. Exploring the energy storage performance of materials is critical to energy storage, and ML accelerates the exploration process. Feng et al. [85] collected over one thousand composite energy storage performance data points from the open literature and utilized ML to analyze and build a predictive model. The prediction accuracies of the RF, SVM, and neural network were 84.1%, 80.9%, and 70.6%, respectively. They then added processed visual information data of the composite into the dataset, resulting in improved prediction accuracies of 91.9%, 68.9%, and 81.6% for the three models, respectively. This demonstrated that the dispersion of the filler in the matrix is an important factor affecting the maximum energy storage density of the composite. The authors also analyzed the weights of each descriptor in the RF model and explored the effects of various parameters on the energy storage of the material. Figure 9 shows the logic diagram of their ML models. Yue et al. [86] utilized the packing dielectric constant, packing size, and packing content as descriptors to predict the energy storage density of polymer matrix composites. High-throughput random breakdown simulations were performed on 504 datasets. The simulation results were then applied as an ML database and combined with classical dielectric prediction equations. They experimentally validated the predictions, including the dielectric constant and breakdown strength. This work provides insights into the design and fabrication of polymer matrix composites with enhanced energy density for applications in capacitive energy storage. Ojin et al. [87] built four traditional ML models and two graph neural network models. Through them, 32,026 heat capacity structures were predicted using a high-precision deep graph attention network. Additionally, the correlation between heat capacity and structure descriptors was inspected. A total of 22 structures were predicted to have high heat capacity, and the results were further validated by DFT analysis. Through the combination of ML and minimal DFT queries, this study provides a path to accelerating the discovery of new thermal energy storage materials.

#### 4.1.4. Structural Health

**Figure 10:**
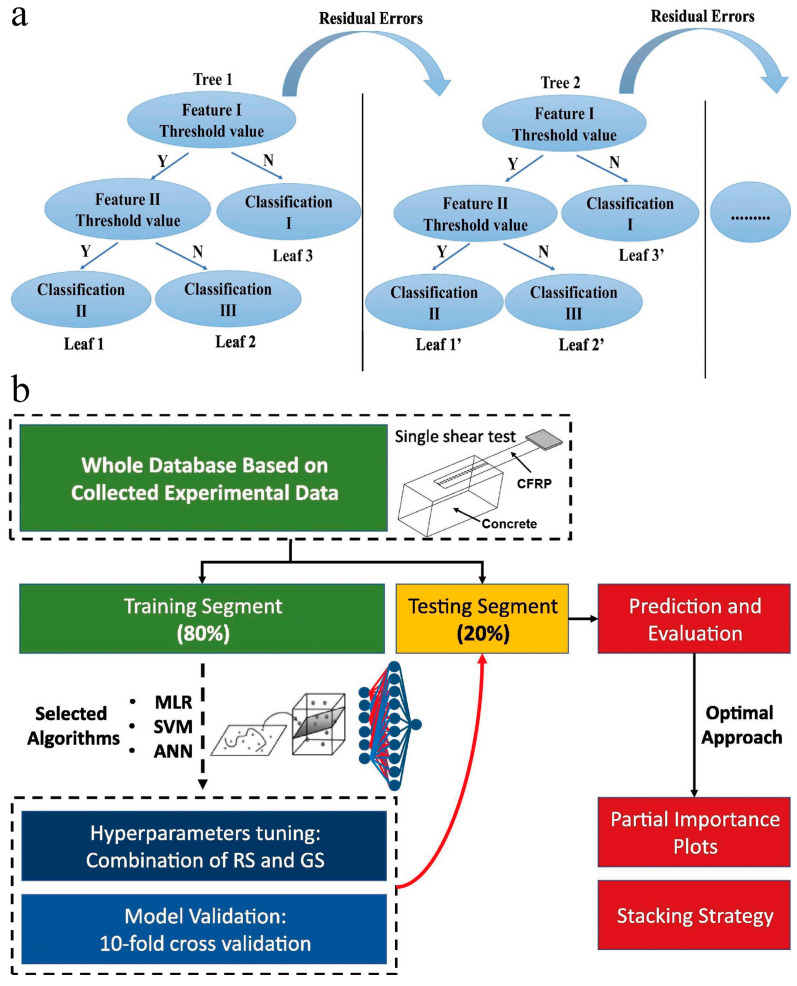
(**a**) Schematic of XGBoost trees, adapted with permission from [91]. (**b**) ML model construction process, adapted with permission from [95].

Structural health monitoring (SHM) utilizes engineering, scientific, and foundational knowledge to prevent damage to property and life. The core of the field of construction informatics is the transmission, processing, and visualization of architectural information, providing effective methods for monitoring structural changes [88,89]. ML provides effective methods for monitoring structural changes. Dang et al. [90] proposed a cloud-based digital twin framework for SHM employing deep learning. The framework consists of physical components, device measurements, and digital models formed by combining different sub-models including mathematical, finite element, and ML sub-models. The data interactions among the physical structure, digital model, and human interventions were enhanced by using cloud computing infrastructure and a user-friendly web application. The feasibility of the framework was demonstrated through case studies of the damage detection of model bridges and real bridge structures utilizing deep learning algorithms, with a high accuracy of 92%. Dong et al. [91] discussed the use of the eXtreme gradient boosting (XGBoost) algorithm for predicting concrete electrical resistivity in SHM (Figure 10a). The proposed XGBoost-algorithm-based prediction model considers all potential influencing factors simultaneously. A database of 800 experimental instances was used to train and test the model. The results showed that the XGBoost model achieved satisfactory predictive performance. The study also identified the importance of curing age and cement content in electrical resistivity measurement results. The XGBoost algorithm was chosen for its high performance, ease of use, and better prediction accuracy than other algorithms. The bond effect between the reinforcement and concrete guarantees the combined action of the two materials. This is a critical factor that affects the mechanical properties of reinforced concrete components and structures, e.g., bearing capacity and ductility [92]. Gao et al. [93] developed a new solution for evaluating the bond strength of an FRP using AI-based models. Two hybrid models, the imperialist competitive algorithm (ICA)-ANN and the artificial bee colony (ABC)-ANN, were designed and compared. The results showed that the ICA-ANN model had a higher predictive ability than the ABC-ANN model. The proposed hybrid models can be used as a suitable substitute for empirical models in evaluating FRP bond strength in concrete samples. Li et al. [94] utilized ML approaches to estimate the bond strength between ultra-high-performance concrete (UHPC) and reinforcing bars. A new database was created by integrating data from multiple published works. Nine ML models, including linear models, tree models, and ANNs, were implemented to train bond strength estimators based on the database. The results showed that the ANN and RF models achieved the highest estimation performances, surpassing empirical formulas. The study also analyzed the relative importance of different factors in determining bond strength. Overall, the research provides a data-driven approach to estimating bond strength and contributes to the understanding of bond performance between UHPC and reinforcing bars. Su et al. [95] applied three ML approaches (multiple linear regression, SVM, and ANN) to predict the interfacial bond strength between FRPs and concrete (Figure 10b). They trained these models using two datasets containing experimental results from single-lap shear tests, employed random search and grid search to find the optimal hyperparameters, and analyzed input variables’ contributions using partial dependence plots. They also developed a stacking strategy to improve prediction accuracy. The results showed that the SVM approach had the best accuracy and efficiency. They concluded that ML methods are feasible and efficient for predicting the bond strength of FRP laminates in reinforced concrete structures.

#### 4.1.5. Nanomaterial Toxicity

**Figure 11:**
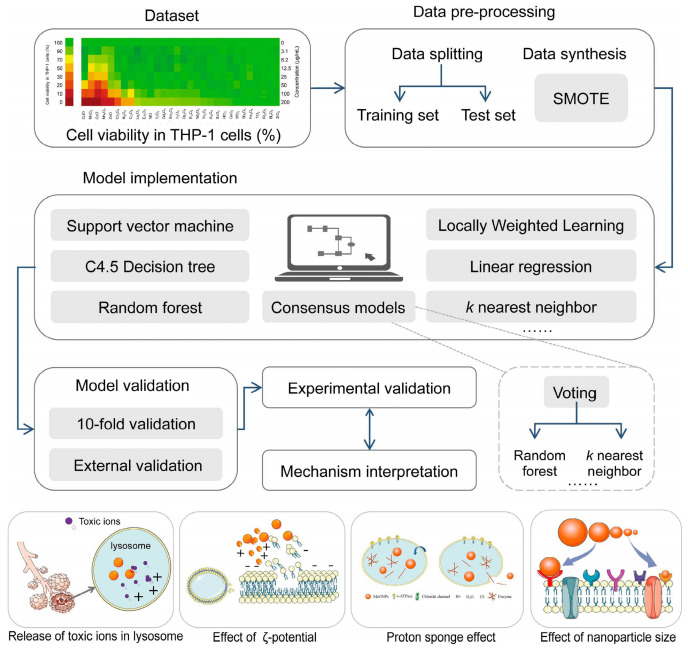
Schematic workflow of data compilation, descriptor generation, machine learning modeling, experimental validation, and mechanism interpretation, reproduced with permission from [97].

It has been proven that ML can be used to identify nanomaterial properties and exposure conditions that influence cellular and organism toxicity, thus providing information required for risk assessment and safe-by-design approaches in the development of new nanomaterials [96]. Huang et al. [97] combined ML with high-throughput in vitro bioassays to develop a model to predict the toxicity of metal oxide nanoparticles to immune cells, as shown in Figure 11. In the training, test, and experimental validation sets, the ML model displayed prediction accuracies of 97%, 96%, and 91%, respectively. ML methods were used to identify features that encode information on immune toxicity. These features are crucial for the scientific design of future experiments and for the accurate depiction of nanotoxicity. According to Gousiadoua et al. [98], advanced ML techniques were applied to create nano quantitative structure–activity relationship (QSAR) tools for modeling the toxicity of metallic and metal oxide nanomaterials, both coated and uncoated, with various core compositions tested on embryonic zebrafish at various dosage concentrations. Based on both computed and experimental descriptors, the scientists identified a set of properties most relevant for assessing nanomaterial toxicity and successfully correlated these properties with zebrafish physiological responses. It has been concluded that for the group of metal and metal oxide nanomaterials, the core chemical composition, concentration, and properties are influenced by the nanomaterial surface and medium composition (such as zeta potential and agglomerate size), which have a significant impact on toxicity, even though the ranking of different variables is subject to variation in the analytical method and data model. Generalized nano-QSAR ensemble models offer a promising framework for predicting the toxicity potential of new nanomaterials. Liu et al. [99] presented a meta-analysis of phytosynthesized silver nanoparticles (AgNPs) with heterogeneous features using DTs and RFs. The researchers found that exposure regime (including the time and dose), plant family, and cell type were the most important predictors for cell viability for green AgNPs. In addition, a discussion of the potential effects of major variables (cell assays, inherent nanoparticle properties, and reaction parameters used in biosynthesis) on AgNP-mediated cytotoxicity and model performance was presented to provide a basis for future research. The findings of this study may assist future studies in improving the design of experiments and the development of virtual models or optimizations of green AgNPs for specific applications.

#### 4.1.6. Adsorption Performance of Nanomaterials

Because of their high surface area, ease of functionalization, and affinity toward a wide range of pollutants, nanomaterials are excellent adsorbents [100]. Moosavi et al. [101] applied four machine learning methods to model dye adsorption on 16 activated carbon adsorbents and determined the relationship between adsorption capacity and activated carbon parameters. The results indicated that agro-waste characteristics (pore volume, surface area, pH, and particle size) contributed 50.7% to the adsorption efficiency. Among the agro-waste characteristics, pore volume and surface area were the most important influencing variables, while particle size had a limited impact. With a hypothetical set of approximately 130,000 structures of metal–organic frameworks (MOFs) with methane and carbon dioxide adsorption data at different pressures, Guo et al. [102] established models for estimating gas adsorption capacities using two deep learning algorithms, multilayer perceptrons (MLPs) and long short-term memory (LSTM) networks. The models were evaluated by performing ten iterations of 10-fold cross-validations and 100 holdout validations. The performance of the MLP and LSTM models was similar with high accuracy of prediction. Those models that predicted gas adsorption at a higher pressure performed better than those that predicted gas adsorption at a lower pressure. In particular, deep learning models were more accurate than RF models reported in the literature when predicting gas adsorption capacities at low pressures. Deep learning algorithms were found to be highly effective in generating models capable of accurately predicting the gas adsorption capacities of MOFs.

### 4.2. Accelerated Materials Synthesis and Design

In addition to being widely utilized for predicting material properties, ML also plays a pivotal role in the synthesis of new materials. During the past few years, ML has made significant progress in the exploration of novel materials, such as highly efficient molecular organic light-emitting diodes [103], low thermal hysteresis shape memory alloys [104], and piezoelectric materials with large electrical strain [105]. The use of ML for materials synthesis not only significantly speeds up novel material discovery but also provides insight into the basic composition changes in materials from big data.

#### 4.2.1. Chalcogenide Materials

Chalcogenide materials can be used in a variety of photovoltaic and energy devices, including light-emitting diodes, photodetectors, and batteries. ML has promoted the development of high-performance chalcogenide materials [106]. Li et al. [107] proposed an ML model based on an RF algorithm for speculating the formation of ABX_3_ and A_2_B′B″X_6_ compound chalcogenides. With geometric and electrical parameters, the RF classification model reached 96.55% accuracy for ABX_3_ samples and 91.83% accuracy for A_2_B′B″X_6_ samples. A total of 241 ABX_3_ chalcogenides with a 95% probability of formation were filtered from 15,999 candidate compounds, and a total of 1131 A_2_B′B″X_6_ chalcogenides with a 99% probability of formation were filtered from 417,835 candidate compounds. The method presented in their work could offer valuable enlightenment for the acceleration of discovering perovskites. Liu et al. [108] used data from 397 ABO_3_ compounds and nine parameters (e.g., tolerance factor and octahedral factor) as input variables for ML. The gradient-enhanced DT obtained by training was compared as the optimal model by 10-fold cross-validation of the average accuracy. A total of 331 chalcogenides were filtered by the model from 891 data points with a classification accuracy of 94.6%. Omprakash et al. [109] compiled a model including organometallic salt chalcogenides to 2D chalcocite and its corresponding band gaps. An ML model for predicting all types of chalcocite band gaps was then trained using a graphical representation learning technique. The model could accurately estimate the band gap within a few milliseconds with an average absolute error of 0.28 eV. Wang et al. [110] applied unsupervised learning to discover quaternary chalcogenide semiconductors (I_2_-II-IV-X_4_) and were successful in screening eight of these materials with good photoconversion efficiency despite a data shortage. This method shortens the material screening cycle and facilitates rapid material discovery.

#### 4.2.2. Catalytic Materials

**Figure 12:**
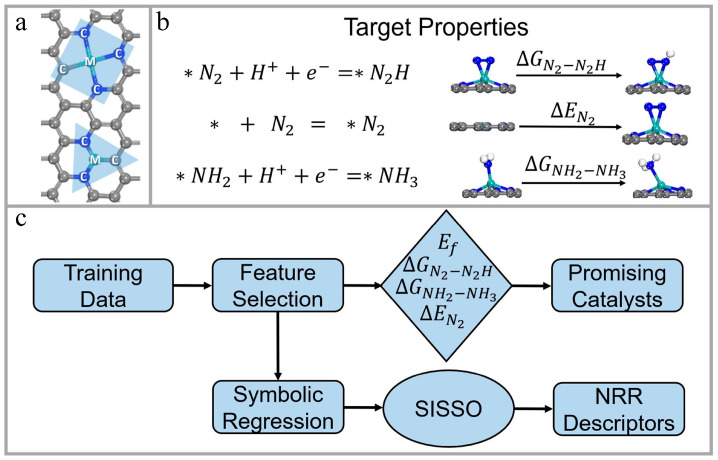
Catalyst structures, target properties, and computational framework. (**a**) Structural representation of three-coordinated and four-coordinated configurations. Letter “M” represents the central metal atom, and letter “C” represents the coordinating atom of M. (**b**) Target properties for describing the N_2_ fixation performance of the catalyst. (**c**) ML screening and descriptor building framework of their work. Reproduced with permission from [112].

In traditional experiments, it is difficult to design efficient catalytic materials in a short time because a clear reaction mechanism is required [111]. ML can rapidly extract the relationship between the structure and performance of catalytic materials and effectively expedite the development process of new catalytic materials. Zhang et al. [112] employed a gradient boosting algorithm to build an ML model. The model utilized four key stability and catalytic features of graphene-loaded single-atom catalysts as targets to find catalytic materials suitable for electro-hydrogenation nitrogen reactions. With this model, a total of 45 catalytic materials with efficient catalytic performance were successfully screened from 1626 samples. The model could be operated for the rapid screening of other electrocatalysts. Figure 12 illustrates their computational framework. Wei et al. [113] developed an ML model, which was applied in a Bayesian optimization framework to obtain molybdenum disulfide (MoS_2_) catalysts with stable hydrogen reaction activity. To explore the structure–property relationship of the samples optimized by the ML technique, nine electrochemical characterizations were performed to verify the results, including SEM, TEM, XRD, and XPS. A strong correlation was found between the structure of the optimized MoS_2_ and its hydrogen evolution reaction performance. Hueffel et al. [114] reported an unsupervised ML workflow that uses only five experimental data points, which could be used to accelerate the recognition of binuclear palladium (Pd) catalysts. Based on their method, some phosphine ligands were successfully predicted and experimentally verified from 348 ligands, including those that had never been synthesized before, which formed binuclear Pd^(I)^ complexes on Pd^(0)^ and Pd^(II)^ species. Their strategy plays an important role in studying the formation mechanisms of Pd catalyst species, as well as the further integration of ML into catalytic research.

#### 4.2.3. Superconducting Materials

**Figure 13:**
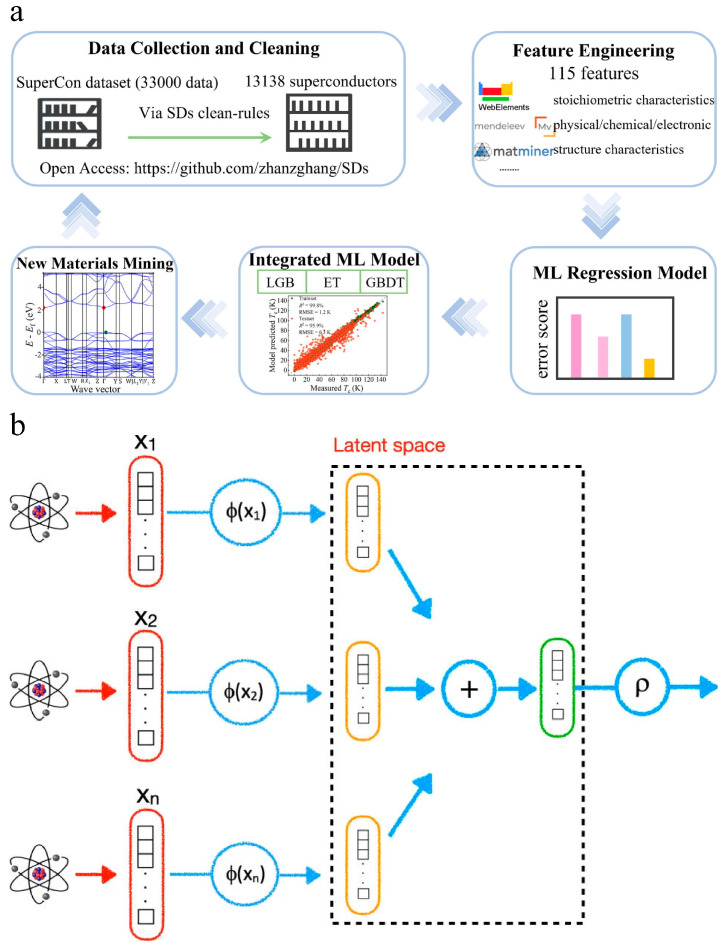
(**a**) Workflow of the integrated model-based ML methods for accurate *T_c_* prediction and new superconductor material mining, adapted with permission from [117]. (**b**) A schematic layout of the DeepSet architecture, adapted with permission from [119].

Superconductivity, intrinsically regulated by finite phonon-coupled electron–electron attractions, has aroused decades of intense research interest in condensed matter physics. The development and prediction of upcoming superconducting materials with high critical temperatures are essential in many applications. ML-guided iterative experimentation may outperform standard high-throughput screening for discovering breakthrough materials in high-temperature superconductors [115,116]. Zhang et al. [117] developed an integrated ML model to accurately and robustly predict the critical temperature (*T_c_*) of superconducting materials (Figure 13a). They used open-source materials data, ML models, and data mining methods to explore the correlation between chemical features and *T_c_* values. The integrated model combined three basic algorithms (gradient boosting decision tree, extra tree, and light gradient boosting machine) to improve the prediction accuracy. The model achieved an *R*^2^ of 95.9% and an RMSE of 6.3 K. The study also identified the importance of various material features in *T_c_* prediction, with thermal conductivity playing a critical role. The integrated model was used to screen out potential superconducting materials with *T_c_* values beyond 50.0 K. This research provides insights for accelerating the exploration of high-*T_c_* superconductors. Roter et al. [118] used ML to predict new superconductors and their critical temperatures. They constructed a database of superconductors and their chemical compositions and applied this information to train ML models. They achieved an *R*^2^ of approximately 0.93, which was comparable to or higher than similar estimates based on other AI techniques. They also discussed factors that limit learning and suggested possible ways to overcome them. The researchers used both unsupervised and supervised ML techniques, including singular value decomposition and KNN, to improve their models’ accuracy. They achieved a classification accuracy of 96.5% and an *R^2^* of approximately 0.93 for predicting critical temperatures. They also employed their models to predict several new superconductors with high critical temperatures. However, the authors noted that incorrect entries in the database can lead to outliers in the predictions. Pereti et al. [119] proposed an ML approach to identify new superconducting materials. They utilized DeepSet technology, which allows them to input the chemical constituents of the compounds without predetermined ordering (Figure 13b). The method was successful in classifying materials as superconducting and quantifying their critical temperature. The trained neural network was then used to search through a mineralogical database for candidates that might be superconducting. Three materials were selected for experimental characterization, and superconductivity was confirmed in two of them. This was the first time a superconducting material was identified using AI methods. The results demonstrated the effectiveness of the DeepSet network in predicting the critical temperatures of superconducting materials.

#### 4.2.4. Nanomaterial Outcome Prediction

Rapid advancements in materials synthesis techniques have led to more and more attention being paid to nanomaterials, including nanocrystals, nanorods, nanoplates, nanoclusters, and nanocrystalline thin films. Materials of this class offer enhanced physical and chemical tunability across a range of systems, including inorganic semiconductors, metals, and molecular crystals. A nanomaterial is defined as a material with a dimension smaller than 100 nanometers in at least one dimension. Unlike bulk materials, nanomaterials possess different physical and chemical properties due to their unique size and shape. This technology has a broad array of application prospects, including the conversion and storage of energy, the restoration of water, medical treatment, and the storage and processing of data.

**Figure 14:**
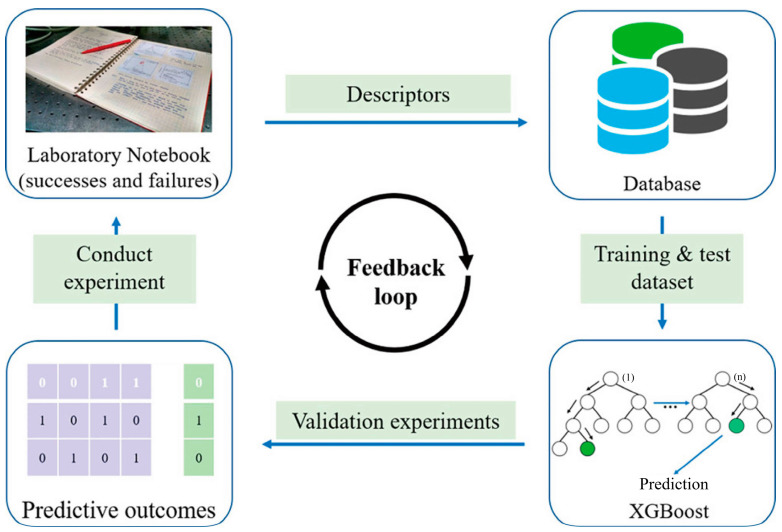
Schematic representation of the working flow when machine learning models are incorporated into the prediction of the crystallization propensity of MONCs, with permission from [120].

Using experimental data, Xie et al. [120] reported the development of an ML-aided method for predicting the crystallization tendency of metal–organic nanocapsules (MONCs). A prediction accuracy of >91% was achieved by using the XGBoost model. Furthermore, they synthesized a set of new crystalline MONCs using the derived features and chemical hypotheses from the XGBoost model. The results of this study demonstrate that ML algorithms can assist chemists in finding the optimal reaction parameters from a large number of experimental parameters more efficiently. Figure 14 shows a schematic representation of the working flow. Pellegrino et al. [121] tuned the TiO_2_ nanoparticle morphology using hydrothermal treatment. In their work, an experimental design was employed to investigate the influence of relevant process parameters on the synthesis outcome, enabling ML methods to develop predictive models. After validation and training, the models were capable of accurately predicting the synthesis outcome in terms of nanoparticle size, polydispersity, and aspect ratio. They presented a synthesis method that allows the continuous and precise control of nanoparticle morphology. This method affords the possibility to tune the aspect ratio over a large range from 1.4 (perfect truncated bipyramids) to 6 (elongated nanoparticles) and a length from 20 to 140 nm.

#### 4.2.5. Nanomaterial Synthesis

Nanomaterial synthesis often involves multiple reagents and interdependent experimental conditions. Each experimental variable’s contribution to the final product is generally determined through trial and error, along with intuition and experience. The process of identifying the most efficient recipe and reaction conditions is therefore time consuming, laborious, and resource intensive [122]. In a recent study, Erick et al. [123] used SVM classification and regression models to predict the synthesis of CsPbBr_3_ nanosheets with controlled layer thicknesses. The SVM classification is shown to accurately predict the likelihood that CsPbBr_3_ synthesis would form a majority population of quantum-confined nanoplatelets. Additionally, SVM regression can be used to determine the average thickness of the synthesis of CsPbBr_3_ nanoplatelets with sub-monolayer accuracy. Epps et al. [124] proposed a method that is based on ML experiment selection and high-efficiency autonomous flow chemistry. The approach utilized SVM regression to predict the thickness of the nanoplatelets and was shown to be accurate and reliable. Using this method, inorganic perovskite quantum dots (QDs) in flow were synthesized autonomously. By using less than 210 mL of starting solutions and without user selection, this method synthesized precision tailored QD compositions within 30 h. This would enable the commercialization of these QDs, as well as their integration into various applications. Furthermore, the method could be used for other types of nanomaterials, such as nanorods and nanowires.

#### 4.2.6. Inverse Design of Nanomaterials

**Figure 15:**
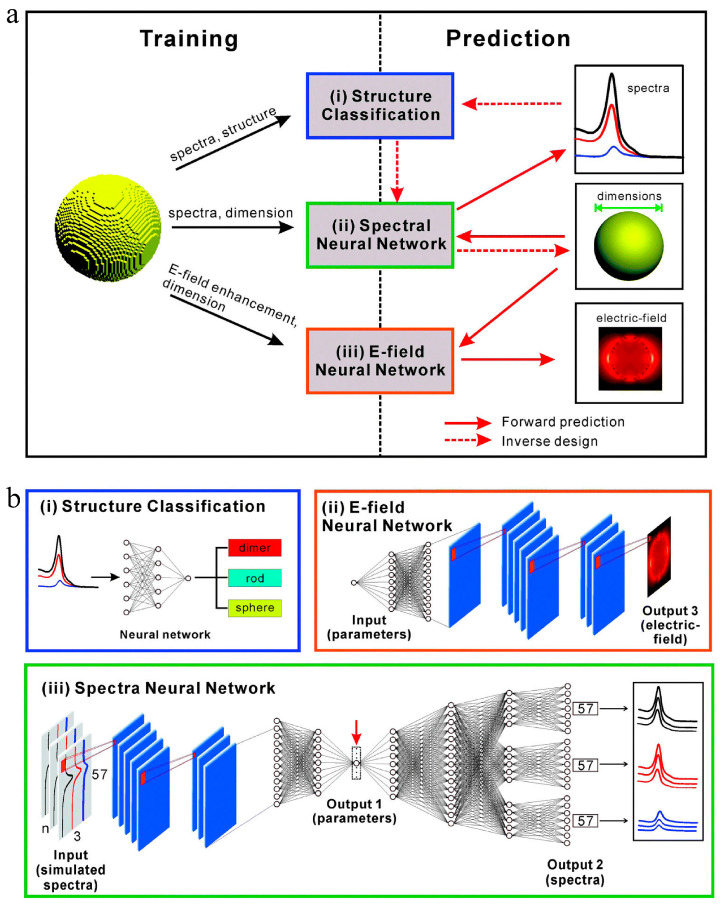
Structures of machine learning models for predicting optical properties and designing nanoparticles. (**a**) Far- and near-field optical data obtained from the finite-difference time-domain (FDTD) simulations were used to train three different machine learning models: far-field spectra and structural information for (i) structure classification, far-field spectra and dimensions for (ii) the spectral DNN, and near-field enhancement maps and dimensions for (iii) the E-field DNN. After training, machine learning models can be used to perform forward prediction and/or inverse design. The solid and dashed red arrows represent the forward prediction and the inverse design process, respectively. (**b**) Detailed architecture of the three machine learning models in Figure 9a, with permission from [128].

As opposed to the direct approach that leads from the chemical space to the desired properties, inverse design starts with desired properties as the “input” and ends with chemical space as the “output” [125]. In the field of nanomaterials, the complexity of inverse design is enhanced by the finite dimensions and variety of shapes, resulting in a larger design space [126]. The inverse design of nanomaterials was quite challenging in the past. The inverse design of nanomaterials could be explored using interpretable relationships between structure and property generated by ML methods. A new inverse design method for metal nanoparticles based on deep learning was proposed and demonstrated by Wang et al. [127]. In comparison to the least squares method, the calculated results indicated that the inverse design method utilizing the back-propagation network had greater adaptability, a smaller minimum error, and can be adjustable based on S parameters. Inverse design systems based on deep learning neural networks may be applied to the inverse design of nanoparticles of different shapes. In another study, Li et al. [126] demonstrated a novel approach to inverse design using multi-target regression methods using RFs. A multi-target regression model was used with a precursory forward structure–property prediction to capture the most important characteristics of a single nanoparticle before the problem was inverted and a number of structural features were simultaneously predicted. A general workflow has been demonstrated on two nanoparticle datasets, and it has the capacity to predict rapid relationships between properties and structures for guiding further research and development without the need for additional optimization or high-throughput sampling. He et al. [128] employed a DNN to establish mappings between the far-field spectra/near-field distribution and dimensional parameters of three different types of plasmonic nanoparticles, including nanospheres, nanorods, and dimers. Through the DNN, both the forward prediction of far-field optical properties and the inverse prediction of nanoparticle dimensional parameters can be accomplished accurately and efficiently. Figure 15 shows the structure of the reported machine learning model for predicting optical properties and designing nanoparticles.

## 5. Conclusions, Challenges, and Prospects

This review discussed the use of machine learning (ML) in the field of materials science for predicting material properties and guiding material synthesis. The review briefly outlined the basic principles of ML and introduced commonly used algorithms and their applications in material screening and property prediction. It also presented the research progress of ML in predicting material properties and guiding material synthesis. The review suggested that ML can greatly reduce computational costs, shorten the development cycle, and improve computational accuracy, making it a promising research approach in novel materials screening and material property prediction.

It is important to note, however, that the following challenges still exist. Most ML algorithms require large amounts of data to work properly. Even for the simplest problems, thousands of examples are desired. Acquiring an effective dataset is critical for the research and implementation of ML in materials science. However, data in materials science are characterized by high acquisition costs, excessive concentration or dispersion, and a lack of uniform processing standards. A dataset with a large amount of data, a uniform distribution, and matching feature parameters is often extremely difficult to obtain. Although material databases have greatly facilitated researchers’ access to data, many published data have not been specified to date. The task of enriching existing databases is challenging. Text mining techniques could be effective in rapidly collecting data scattered in the literature. This approach could greatly enhance existing databases and create specialized databases.

The selection of features significantly affects the accuracy of ML models. Currently, the use of manual feature engineering to filter features is often influenced by the researcher’s experience and intuition. This approach may overlook some significant features. In contrast, automated feature engineering automatically constructs new candidate features from the data and selects the most appropriate features for model training, which could effectively solve the current dilemma.

ML methods cannot replace traditional computational and experimental studies. Although ML methods have shown remarkable promise in guiding the synthesis of novel materials and predicting material properties, they are still mostly “black boxes” [108]. The predicted results still need to be experimentally verified and the underlying physicochemical laws still need to be studied in depth. Therefore, ML can only perform some exploratory tasks at present. With further improvement of theories and methods, however, ML might eventually replace traditional experimental research by providing novel ideas and research methods for the field of materials science. The application of ML in the field of materials science and engineering is just the beginning, and its potential is endless in the future.

## Figures and Tables

**Table 1:** An overview of some databases in material science.

Database	Website	Brief Introduction
AFLOW	http://www.aflowlib.org/ (accessed on 17 July 2023)	A globally available database of 3,530,330 material compounds with over 734,308,640 calculated properties and growing.
Crystallography Open Database (COD)	http://www.crystallography.net/ (accessed on 17 July 2023)	Open-access collection of crystal structures of organic, inorganic, metal–organic compounds and minerals, excluding biopolymers.
Cambridge Structural Database (CSD)	https://www.ccdc.cam.ac.uk/ (accessed on 17 July 2023)	The world’s largest database of small-molecule organic and metal–organic crystal structure data, now at over 1.2 million structures.
Inorganic Crystal Structure Database (ICSD)	http://cds.dl.ac.uk/ (accessed on 17 July 2023)	A comprehensive collection of crystal structure information for non-organic compounds, including inorganics, ceramics, minerals, and metals, covers the literature from 1915 to the present and contains over 60,000 entries on the crystal structure of in-organic materials.
Materials Project	https://materialsproject.org/ (accessed on 17 July 2023)	A database containing 154,718 materials, 4351 intercalation electrodes, and 172,874 molecules.
Open Quantum Materials Database (OQMD)	http://oqmd.org/ (accessed on 17 July 2023)	The OQMD is a database of DFT calculated thermodynamic and structural properties of 1,022,603 materials.

## Abbreviations

ABC	Artificial bee colony
AFLOW	Automatic Flow
AgNP	Silver nanoparticle
AI	Artificial intelligence
ANN	Artificial neural network
CNN	Convolutional neural network
COD	Crystallography Open Database
CSD	Cambridge Structural Database
DBM	Deep Boltzmann machine
DBN	Deep belief network
DFT	Density functional theory
DNN	Deep neural network
DT	Decision tree
FDTD	Finite-difference time-domain
FRP	Fiber-reinforced polymer
GAN	Generative adversarial network
GBRT	Gradient boosted regression tree
GGA	Generalized gradient approximation
GLOnet	Global optimization network
HEA	High-entropy alloy
HHV	Higher heating value
ICA	Imperialist competitive algorithm
ICSD	Inorganic Crystal Structure Database
JAMIP	Jilin Artificial-intelligence aided Materials-design Integrated Package
KNN	K-nearest neighbor
LSTM	Long short-term memory
MDN	Mixture density network
ML	Machine learning
MLP	Multilayer perceptron
MOF	Metal–organic framework
MONC	Metal–organic nanocapsule
NMR	Nuclear magnetic resonance
OMDB	Organic Materials Database
OQMD	Open Quantum Materials Database
QD	Quantum dot
QSAR	Quantitative structure–activity relationship
R-CNN	Region-based CNN
ResNet	Residual network
RF	Random forest
RMSE	Root mean square error
RNN	Recurrent neural network
SEM	Scanning electron microscope
SHM	Structural health monitoring
SMILE	Simplified molecular input line entry
SVM	Support vector machine
SVR	Support vector regression
TEM	Transmission electron microscope
TGNN	Tupleswise graph neural network
UHPC	Ultra-high-performance concrete
XGBoost	eXtreme gradient boosting
XPS	X-ray photoelectron spectroscopy
XRD	X-ray diffraction
μ-PTAMAM	Micro-plasma transfer arc metal additive manufacturing
